# Supplementary material for: Risks of Stroke and Transient Cerebral Ischemia up to 4 Years Post-SARS-CoV-2 Infection in Large Diverse Urban Population in the Bronx
Source: Diagnostics (Basel). 2025 Dec 13;15(24):3183. doi: 10.3390/diagnostics15243183 (PMC12731514; doi:10.3390/diagnostics15243183)
Supplement: Supplementary file 1 [file diagnostics-15-03183-s001.zip › diagnostics-4004216_supplementary file S1.pdf]

Supplemental File S1

CONCEPT_ID	CONCEPT_NAME	CONCEPT_CODE	VOCABULARY_ID	DOMAIN_ID
Stroke				
374384	Cerebral ischemia	287731003	SNOMED	Condition
381591	Cerebrovascular disease	62914000	SNOMED	Condition
443454	Cerebral infarction	432504007	SNOMED	Condition
4046360	Lacunar infarction	230698000	SNOMED	Condition
4108356	Cerebral infarction due to embolism of cerebral arteries	195190007	SNOMED	Condition
4110190	Cerebral infarction due to embolism of precerebral arteries	195186005	SNOMED	Condition
4110192	Cerebral infarction due to thrombosis of cerebral arteries	195189003	SNOMED	Condition
4111710	Brainstem stroke syndrome	195212005	SNOMED	Condition
4111711	Cerebellar stroke syndrome	195213000	SNOMED	Condition
4111714	Cerebral infarction due to cerebral venous thrombosis, non-pyogenic	195230003	SNOMED	Condition
42535227	Acute cerebral ischemia	16218291000119100	SNOMED	Condition
45772786	Cerebral infarction due to embolism of middle cerebral artery	705128004	SNOMED	Condition
46270031	Cerebral infarction due to occlusion of precerebral artery	125081000119106	SNOMED	Condition
46273649	Cerebral infarction due to occlusion of basilar artery	34181000119102	SNOMED	Condition
Transient cerebral ischemia				
373503	Transient cerebral ischemia	266257000	SNOMED	Condition
COVID-19 infection				
706163	SARS-CoV-2 (COVID-19) RNA [Presence] in Respiratory specimen by NAA with probe detection	94500-6	LOINC	Measurement
706170	SARS-CoV-2 (COVID-19) RNA [Presence] in Unspecified specimen by NAA with probe detection	94309-2	LOINC	Measurement

Vaccination				
739906	SARS-COV-2 (COVID-19) vaccine, vector - Ad26 100000000000 UNT/ML Injectable Suspension	2479835	RxNorm	Drug
35894915	COVID-19 vaccine	OMOP5042939	RxNorm Extension	Drug
37003435	SARS-CoV-2 (COVID-19) vaccine, mRNA spike protein Injectable Suspension	2468234	RxNorm	Drug
37003436	SARS-CoV-2 (COVID-19) vaccine, mRNA-BNT162b2 0.1 MG/ML Injectable Suspension	2468235	RxNorm	Drug
37003518	SARS-CoV-2 (COVID-19) vaccine, mRNA-1273 0.2 MG/ML Injectable Suspension	2470234	RxNorm	Drug
Cardiovascular disease				
44782718	Acute combined systolic and diastolic heart failure	153931000119109	SNOMED	Condition
40481042	Acute diastolic heart failure	443343001	SNOMED	Condition
319844	Acute ischemic heart disease	413439005	SNOMED	Condition
44782733	Acute on chronic combined systolic and diastolic heart failure	153951000119103	SNOMED	Condition
40481043	Acute on chronic diastolic heart failure	443344007	SNOMED	Condition
37309625	Acute on chronic right-sided congestive heart failure	16838951000119100	SNOMED	Condition
40480602	Acute on chronic systolic heart failure	443253003	SNOMED	Condition
4233424	Acute right-sided heart failure	359617009	SNOMED	Condition
40480603	Acute systolic heart failure	443254009	SNOMED	Condition
316427	Aneurysm of coronary vessels	50570003	SNOMED	Condition
36712983	Angina co-occurrent and due to coronary arteriosclerosis	15960141000119102	SNOMED	Condition
321318	Angina pectoris	194828000	SNOMED	Condition
42537729	Aortocoronary bypass graft present	737276005	SNOMED	Condition
443551	Apraxia due to cerebrovascular accident	428668000	SNOMED	Condition
43021857	Arteriosclerosis of autologous arterial coronary artery bypass graft	285141000119106	SNOMED	Condition
40482638	Arteriosclerosis of autologous vein coronary artery bypass graft	442224005	SNOMED	Condition

443563	Arteriosclerosis of coronary artery bypass graft	429673002	SNOMED	Condition
40481132	Arteriosclerosis of coronary artery bypass graft of transplanted heart	444855007	SNOMED	Condition
40482655	Arteriosclerosis of nonautologous coronary artery bypass graft	442240008	SNOMED	Condition
43531622	Ataxia as sequela of cerebrovascular disease	29941000119105	SNOMED	Condition
764123	Atherosclerosis of coronary artery without angina pectoris	451041000124103	SNOMED	Condition
4242669	Biventricular congestive heart failure	92506005	SNOMED	Condition
4111710	Brainstem stroke syndrome	195212005	SNOMED	Condition
321042	Cardiac arrest	410429000	SNOMED	Condition
4309332	Cardiac arrest as a complication of care	213213007	SNOMED	Condition
4172822	Cardiac arrest due to cardiac disorder	423191000	SNOMED	Condition
46274066	Cardiac arrest due to incomplete miscarriage	10811961000119109	SNOMED	Condition
46269812	Cardiac arrest due to miscarriage	10760181000119109	SNOMED	Condition
4311273	Cardiac arrest during AND/OR resulting from a procedure	86152005	SNOMED	Condition
4111711	Cerebellar stroke syndrome	195213000	SNOMED	Condition
4110189	Cerebral infarct due to thrombosis of precerebral arteries	195185009	SNOMED	Condition
443454	Cerebral infarction	432504007	SNOMED	Condition
4111714	Cerebral infarction due to cerebral venous thrombosis, non-pyogenic	195230003	SNOMED	Condition
4108356	Cerebral infarction due to embolism of cerebral arteries	195190007	SNOMED	Condition
45772786	Cerebral infarction due to embolism of middle cerebral artery	705128004	SNOMED	Condition
4110190	Cerebral infarction due to embolism of precerebral arteries	195186005	SNOMED	Condition
46273649	Cerebral infarction due to occlusion of basilar artery	34181000119102	SNOMED	Condition
46270031	Cerebral infarction due to occlusion of precerebral artery	125081000119106	SNOMED	Condition
4110192	Cerebral infarction due to thrombosis of cerebral arteries	195189003	SNOMED	Condition
45767658	Cerebral infarction due to thrombosis of middle cerebral artery	705130002	SNOMED	Condition

381591	Cerebrovascular disease	62914000	SNOMED	Condition
44782719	Chronic combined systolic and diastolic heart failure	153941000119100	SNOMED	Condition
4229440	Chronic congestive heart failure	88805009	SNOMED	Condition
40479576	Chronic diastolic heart failure	441530006	SNOMED	Condition
315286	Chronic ischemic heart disease	413838009	SNOMED	Condition
4014159	Chronic right-sided heart failure	10335000	SNOMED	Condition
40479192	Chronic systolic heart failure	441481004	SNOMED	Condition
36712779	Chronic total occlusion of coronary artery	117051000119103	SNOMED	Condition
319835	Congestive heart failure	42343007	SNOMED	Condition
37312532	Coronary arteriosclerosis in artery of transplanted heart	792842004	SNOMED	Condition
42537730	Coronary artery graft present	737278006	SNOMED	Condition
4127089	Coronary artery spasm	23687008	SNOMED	Condition
40481919	Coronary atherosclerosis	443502000	SNOMED	Condition
4108215	Coronary thrombosis not resulting in myocardial infarction	194821006	SNOMED	Condition
443587	Diastolic heart failure	418304008	SNOMED	Condition
37115756	Dissection of coronary artery	732230001	SNOMED	Condition
443465	Dysphagia as a late effect of cerebrovascular accident	426033005	SNOMED	Condition
316139	Heart failure	84114007	SNOMED	Condition
44782781	Hemiplegia and/or hemiparesis following stroke	48601000119107	SNOMED	Condition
4004279	High output heart failure	10091002	SNOMED	Condition
439696	Hypertensive heart and renal disease with (congestive) heart failure	194779001	SNOMED	Condition
319034	Hypertensive heart disease without congestive heart failure	60899001	SNOMED	Condition
444101	Hypertensive heart failure	46113002	SNOMED	Condition
40479572	Infarct of cerebrum due to iatrogenic cerebrovascular accident	441526008	SNOMED	Condition
4219010	Juvenile myopathy, encephalopathy, lactic acidosis AND stroke	39925003	SNOMED	Condition
439846	Left heart failure	85232009	SNOMED	Condition
43020458	Mechanical breakdown of coronary artery bypass graft	285951000119105	SNOMED	Condition
432499	Mechanical complication due to coronary bypass graft	78717006	SNOMED	Condition
443525	Monoplegia of dominant upper limb as a late effect of cerebrovascular accident	427065003	SNOMED	Condition

40480946	Monoplegia of nondominant lower limb as a late effect of cerebrovascular accident	441894009	SNOMED	Condition
40482266	Monoplegia of nondominant upper limb as a late effect of cerebrovascular accident	442181008	SNOMED	Condition
40481842	Monoplegia of upper limb as late effect of cerebrovascular disease	442097001	SNOMED	Condition
4106274	Neonatal cardiac arrest	180906006	SNOMED	Condition
4159152	Neonatal stroke	371121002	SNOMED	Condition
372654	Paralytic syndrome as late effect of stroke	425882004	SNOMED	Condition
443609	Paralytic syndrome of dominant side as late effect of stroke	430959006	SNOMED	Condition
443599	Paralytic syndrome of nondominant side as late effect of stroke	430947007	SNOMED	Condition
43530742	Paralytic syndrome on one side of the body as late effect of cerebrovascular accident	361000119103	SNOMED	Condition
4198141	Post infarct angina	314116003	SNOMED	Condition
315296	Preinfarction syndrome	4557003	SNOMED	Condition
40482301	Residual cognitive deficit as late effect of cerebrovascular accident	442212003	SNOMED	Condition
4195785	Right heart failure secondary to left heart failure	44313006	SNOMED	Condition
4124683	Silent myocardial ischemia	233823002	SNOMED	Condition
40481354	Speech and language deficit as late effect of cerebrovascular accident	441960006	SNOMED	Condition
443580	Systolic heart failure	417996009	SNOMED	Condition
433195	Transient arterial retinal occlusion	87224000	SNOMED	Condition
373503	Transient cerebral ischemia	266257000	SNOMED	Condition
36712982	Unstable angina co-occurrent and due to coronary arteriosclerosis	15960061000119102	SNOMED	Condition
44782753	Weakness as a late effect of stroke	148871000119109	SNOMED	Condition
43530744	Weakness of face muscles as sequela of stroke	40161000119102	SNOMED	Condition
Type-2 Diabetes				
4196141	Arthropathy due to type 2 diabetes mellitus	314903002	SNOMED	Condition
4175440	Autonomic neuropathy due to diabetes mellitus	50620007	SNOMED	Condition

37016768	Autonomic neuropathy due to type 2 diabetes mellitus	712883005	SNOMED	Condition
376979	Cataract due to diabetes mellitus	43959009	SNOMED	Condition
4221495	Cataract due to diabetes mellitus type 2	420756003	SNOMED	Condition
442793	Complication due to diabetes mellitus	74627003	SNOMED	Condition
201820	Diabetes mellitus	73211009	SNOMED	Condition
4058243	Diabetes mellitus during pregnancy, childbirth and the puerperium	199223000	SNOMED	Condition
45757129	Diabetes mellitus in mother complicating childbirth	10754881000119104	SNOMED	Condition
4008576	Diabetes mellitus without complication	111552007	SNOMED	Condition
4009303	Diabetic ketoacidosis without coma	111556005	SNOMED	Condition
443767	Disorder of eye due to diabetes mellitus	25093002	SNOMED	Condition
443733	Disorder of eye due to type 2 diabetes mellitus	422099009	SNOMED	Condition
192279	Disorder of kidney due to diabetes mellitus	127013003	SNOMED	Condition
443730	Disorder of nervous system due to diabetes mellitus	422088007	SNOMED	Condition
376065	Disorder of nervous system due to type 2 diabetes mellitus	421326000	SNOMED	Condition
43530690	Foot ulcer due to type 2 diabetes mellitus	1521000119100	SNOMED	Condition
4226354	Gangrene due to diabetes mellitus	422275004	SNOMED	Condition
4222876	Gangrene due to type 2 diabetes mellitus	421631007	SNOMED	Condition
37016349	Hyperglycemia due to type 2 diabetes mellitus	368051000119109	SNOMED	Condition
4226238	Hyperosmolar coma due to diabetes mellitus	422126006	SNOMED	Condition
201530	Hyperosmolar coma due to type 2 diabetes mellitus	190331003	SNOMED	Condition
4029423	Hypoglycemia due to diabetes mellitus	237633009	SNOMED	Condition
45757363	Hypoglycemia due to type 2 diabetes mellitus	120731000119103	SNOMED	Condition
4226798	Hypoglycemic coma due to diabetes mellitus	421725003	SNOMED	Condition

36714116	Hypoglycemic coma due to type 2 diabetes mellitus	719216001	SNOMED	Condition
4095288	Ketoacidotic coma due to diabetes mellitus	26298008	SNOMED	Condition
4228443	Ketoacidotic coma due to type 2 diabetes mellitus	421847006	SNOMED	Condition
37110593	Lesion of skin due to diabetes mellitus	724876003	SNOMED	Condition
4191611	Lumbosacral radiculoplexus neuropathy due to diabetes mellitus	39058009	SNOMED	Condition
4140466	Lumbosacral radiculoplexus neuropathy due to type 2 diabetes mellitus	427027005	SNOMED	Condition
45770830	Macular edema and retinopathy due to type 2 diabetes mellitus	97331000119101	SNOMED	Condition
380097	Macular edema due to diabetes mellitus	312912001	SNOMED	Condition
35626068	Macular edema of left eye due to diabetes mellitus	769218003	SNOMED	Condition
35626067	Macular edema of right eye due to diabetes mellitus	769217008	SNOMED	Condition
378743	Mild nonproliferative retinopathy due to diabetes mellitus	312903003	SNOMED	Condition
45757435	Mild nonproliferative retinopathy due to type 2 diabetes mellitus	138911000119106	SNOMED	Condition
35626039	Mild nonproliferative retinopathy of left eye due to diabetes mellitus	769184004	SNOMED	Condition
35626038	Mild nonproliferative retinopathy of right eye due to diabetes mellitus	769183005	SNOMED	Condition
377552	Moderate nonproliferative retinopathy due to diabetes mellitus	312904009	SNOMED	Condition
45770881	Moderate nonproliferative retinopathy due to type 2 diabetes mellitus	138921000119104	SNOMED	Condition
4222415	Mononeuropathy due to type 2 diabetes mellitus	420436000	SNOMED	Condition
4114427	Neuropathic arthropathy due to diabetes mellitus	201724008	SNOMED	Condition
43531563	Neuropathic arthropathy due to type 2 diabetes mellitus	781000119106	SNOMED	Condition
4044391	Neuropathy due to diabetes mellitus	230572002	SNOMED	Condition
43530656	Nonproliferative retinopathy due to type 2 diabetes mellitus	1551000119108	SNOMED	Condition

4131908	Peripheral angiopathy due to diabetes mellitus	127014009	SNOMED	Condition
443729	Peripheral circulatory disorder due to type 2 diabetes mellitus	422166005	SNOMED	Condition
321822	Peripheral vascular disorder due to diabetes mellitus	421895002	SNOMED	Condition
376112	Polyneuropathy due to diabetes mellitus	49455004	SNOMED	Condition
37017432	Polyneuropathy due to type 2 diabetes mellitus	713706002	SNOMED	Condition
45757079	Pre-existing diabetes mellitus in mother complicating childbirth	106281000119103	SNOMED	Condition
43531007	Pre-existing diabetes mellitus in pregnancy	609563008	SNOMED	Condition
4063043	Pre-existing type 2 diabetes mellitus	199230006	SNOMED	Condition
43531010	Pre-existing type 2 diabetes mellitus in pregnancy	609567009	SNOMED	Condition
380096	Proliferative retinopathy due to diabetes mellitus	59276001	SNOMED	Condition
43530685	Proliferative retinopathy due to type 2 diabetes mellitus	1501000119109	SNOMED	Condition
443731	Renal disorder due to type 2 diabetes mellitus	420279001	SNOMED	Condition
4174977	Retinopathy due to diabetes mellitus	4855003	SNOMED	Condition
4029420	Severe hyperglycemia due to diabetes mellitus	237621004	SNOMED	Condition
376114	Severe nonproliferative retinopathy due to diabetes mellitus	312905005	SNOMED	Condition
35626044	Severe nonproliferative retinopathy of left eye due to diabetes mellitus	769188001	SNOMED	Condition
35626043	Severe nonproliferative retinopathy of right eye due to diabetes mellitus	769187006	SNOMED	Condition
4290822	Severe nonproliferative retinopathy with clinically significant macular edema due to diabetes mellitus	399872003	SNOMED	Condition
4266637	Severe nonproliferative retinopathy without macular edema due to diabetes mellitus	399873008	SNOMED	Condition
4227657	Skin ulcer due to diabetes mellitus	422183001	SNOMED	Condition
4338901	Traction detachment of retina due to diabetes mellitus	232023006	SNOMED	Condition
45773064	Traction detachment of retina due to type 2 diabetes mellitus	82541000119100	SNOMED	Condition

201826	Type 2 diabetes mellitus	44054006	SNOMED	Condition
4099651	Type 2 diabetes mellitus with ulcer	190389009	SNOMED	Condition
4193704	Type 2 diabetes mellitus without complication	313436004	SNOMED	Condition
CKD				
45768812	Anemia in chronic kidney disease	707323002	SNOMED	Condition
46271022	Chronic kidney disease	709044004	SNOMED	Condition
44782429	Chronic kidney disease due to hypertension	104931000119100	SNOMED	Condition
43531578	Chronic kidney disease due to type 2 diabetes mellitus	771000119108	SNOMED	Condition
443614	Chronic kidney disease stage 1	431855005	SNOMED	Condition
443601	Chronic kidney disease stage 2	431856006	SNOMED	Condition
443597	Chronic kidney disease stage 3	433144002	SNOMED	Condition
45763854	Chronic kidney disease stage 3A	700378005	SNOMED	Condition
45763855	Chronic kidney disease stage 3B	700379002	SNOMED	Condition
443612	Chronic kidney disease stage 4	431857002	SNOMED	Condition
443611	Chronic kidney disease stage 5	433146000	SNOMED	Condition
193782	End-stage renal disease	46177005	SNOMED	Condition
4127554	Failed renal transplant	236583003	SNOMED	Condition
44784621	Hypertensive heart and chronic kidney disease	8501000119104	SNOMED	Condition
443919	Hypertensive renal failure	49220004	SNOMED	Condition
43021985	Infection associated with peritoneal dialysis catheter	473069007	SNOMED	Condition
43021418	Leakage of peritoneal dialysis catheter	473190001	SNOMED	Condition
4070976	Mechanical complication of dialysis catheter	17778006	SNOMED	Condition
440302	Mechanical complication of peritoneal dialysis catheter	431028002	SNOMED	Condition
4126451	Migration of peritoneal dialysis catheter	236563004	SNOMED	Condition
44782924	Misplacement of hemodialysis catheter	698937002	SNOMED	Condition
45757356	Pre-existing hypertensive chronic kidney disease in mother complicating pregnancy	118781000119108	SNOMED	Condition
45757137	Pre-existing hypertensive heart and chronic kidney disease in mother complicating childbirth	10757401000119104	SNOMED	Condition

45757139	Pre-existing hypertensive heart and chronic kidney disease in mother complicating pregnancy	10757481000119107	SNOMED	Condition
197921	Renal osteodystrophy	16726004	SNOMED	Condition
4128369	Renal transplant rejection	236570004	SNOMED	Condition
COPD				
257004	Acute exacerbation of chronic obstructive airways disease	195951007	SNOMED	Condition
4286497	Centriacinar emphysema	68328006	SNOMED	Condition
255841	Chronic bronchitis	63480004	SNOMED	Condition
255573	Chronic obstructive lung disease	13645005	SNOMED	Condition
4110056	Chronic obstructive pulmonary disease with acute lower respiratory infection	196001008	SNOMED	Condition
4112826	Mixed simple and mucopurulent chronic bronchitis	195953005	SNOMED	Condition
257905	Mucopurulent chronic bronchitis	74417001	SNOMED	Condition
4177944	Panacinar emphysema	4981000	SNOMED	Condition
261325	Pulmonary emphysema	87433001	SNOMED	Condition
261889	Simple chronic bronchitis	61937009	SNOMED	Condition
Asthma				
45771045	Acute exacerbation of asthma	708038006	SNOMED	Condition
46270082	Acute exacerbation of mild persistent asthma	135181000119109	SNOMED	Condition
46273487	Acute exacerbation of moderate persistent asthma	135171000119106	SNOMED	Condition
45769438	Acute severe exacerbation of asthma	708090002	SNOMED	Condition
45769352	Acute severe exacerbation of mild persistent asthma	707981009	SNOMED	Condition
45769351	Acute severe exacerbation of moderate persistent asthma	707980005	SNOMED	Condition
45769350	Acute severe exacerbation of severe persistent asthma	707979007	SNOMED	Condition
37116845	Acute severe refractory exacerbation of asthma	733858005	SNOMED	Condition
317009	Asthma	195967001	SNOMED	Condition
313236	Cough variant asthma	409663006	SNOMED	Condition
4279553	Eosinophilic asthma	367542003	SNOMED	Condition
4138760	Exacerbation of intermittent asthma	425969006	SNOMED	Condition

4146581	Mild intermittent asthma	427679007	SNOMED	Condition
45768910	Uncomplicated asthma	707444001	SNOMED	Condition
45768963	Uncomplicated mild persistent asthma	707511009	SNOMED	Condition
45768964	Uncomplicated moderate persistent asthma	707512002	SNOMED	Condition
45768965	Uncomplicated severe persistent asthma	707513007	SNOMED	Condition
Hypertension				
312902	Benign intracranial hypertension	68267002	SNOMED	Condition
44782429	Chronic kidney disease due to hypertension	104931000119100	SNOMED	Condition
4313767	Chronic peripheral venous hypertension	423674003	SNOMED	Condition
44782715	Chronic peripheral venous hypertension with lower extremity complication	153811000119105	SNOMED	Condition
320128	Essential hypertension	59621000	SNOMED	Condition
42538946	Hypertension complicating pregnancy	82771000119102	SNOMED	Condition
4110948	Hypertension secondary to endocrine disorder	194788005	SNOMED	Condition
4118910	Maternal hypertension	288250001	SNOMED	Condition
4071202	Neonatal hypertension	206596003	SNOMED	Condition
381290	Ocular hypertension	4210003	SNOMED	Condition
192680	Portal hypertension	34742003	SNOMED	Condition
45757788	Postpartum pregnancy-induced hypertension	40521000119100	SNOMED	Condition
321074	Pre-existing hypertension complicating pregnancy, childbirth and puerperium	199005000	SNOMED	Condition
4311246	Pre-existing hypertension in obstetric context	86041002	SNOMED	Condition
4057979	Pre-existing secondary hypertension complicating pregnancy, childbirth and puerperium	199008003	SNOMED	Condition
4167493	Pregnancy-induced hypertension	48194001	SNOMED	Condition
443771	Renal hypertension	28119000	SNOMED	Condition
317895	Renovascular hypertension	123799005	SNOMED	Condition
319826	Secondary hypertension	31992008	SNOMED	Condition
