# Supplementary material for: Risks of Stroke and Transient Cerebral Ischemia up to 4 Years Post-SARS-CoV-2 Infection in Large Diverse Urban Population in the Bronx
Source: Diagnostics (Basel). 2025 Dec 13;15(24):3183. doi: 10.3390/diagnostics15243183 (PMC12731514; doi:10.3390/diagnostics15243183)
Supplement: Supplementary file 1 [file diagnostics-15-03183-s001.zip › diagnostics-4004216_supplementary file S2.pdf]

Supplementary File S2

A subgroup analysis stratified by age, sex, race, ethnicity, ZIP code median income quartile, insurance coverage, comorbidity presence, and COVID-19 vaccination status

Supplemental Figure S1. Cox-proportional hazard ratio model results on stratified data for outcome ischemic stroke. Hazard ratio is adjusted for other covariates. **A)** Hospitalized Covid positive vs Covid Negative **B)** Non-hospitalized Covid Positive vs Covid Negative

A)

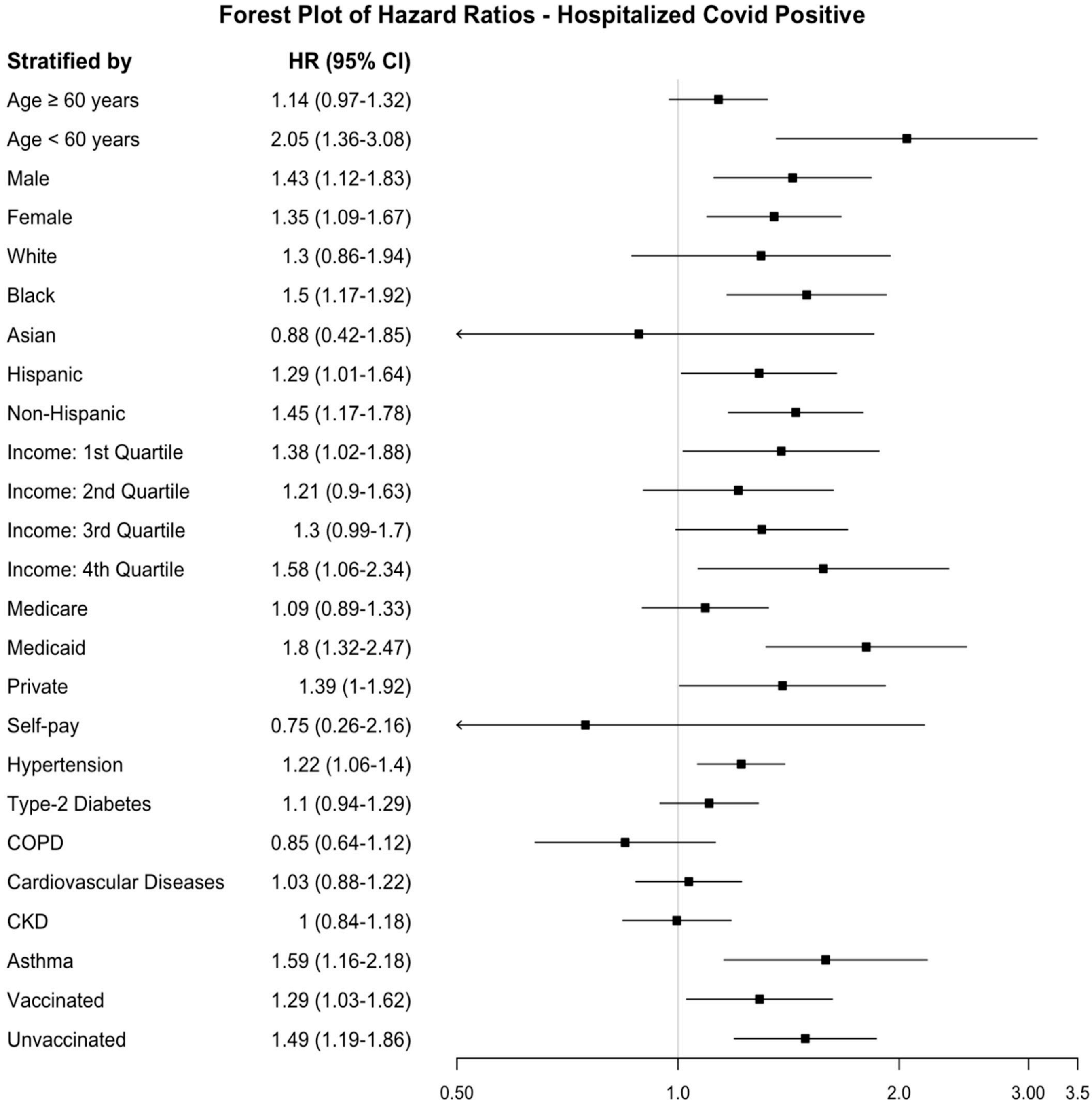

B)

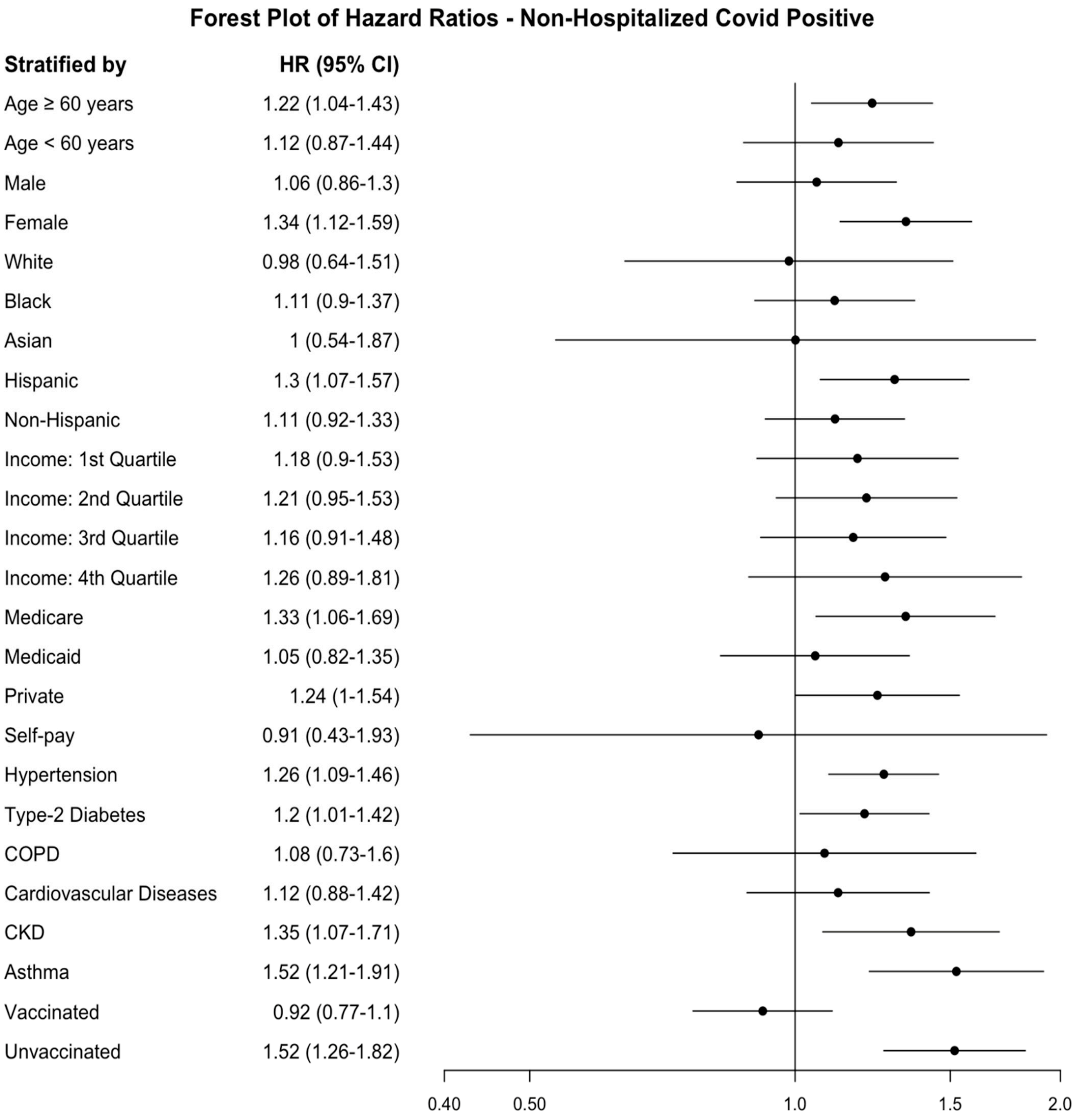

Supplemental Figure S2. Cox-proportional hazard ratio model results on stratified data for outcome Transient cerebral ischemia. Hazard ratio is adjusted for other covariates. **A)** Hospitalized Covid positive vs Covid Negative **B)** Non-hospitalized Covid Positive vs Covid Negative

A)

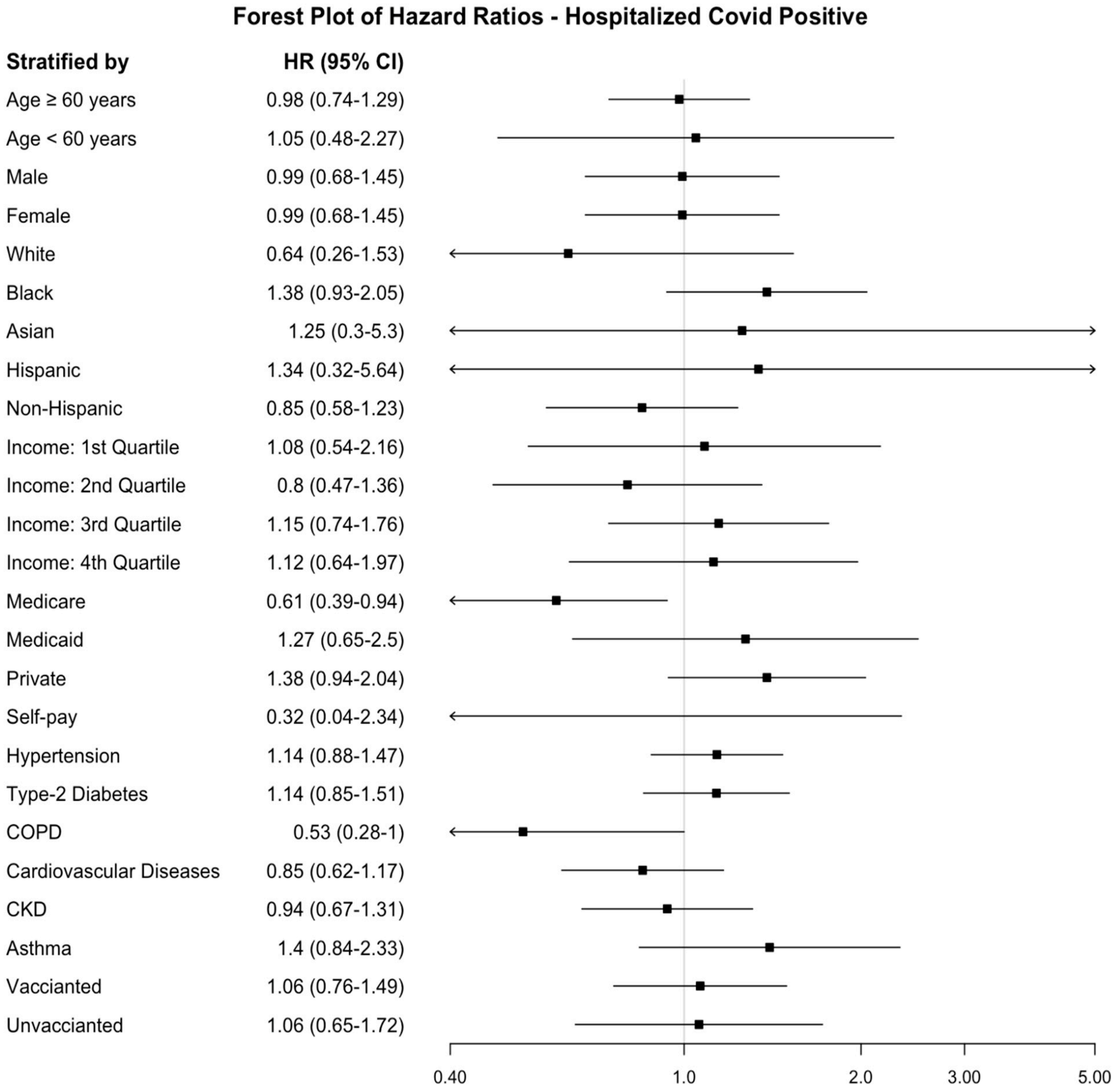

Forest Plot of Hazard Ratios - Hospitalized Covid Positive
Stratified by
HR (95% CI)
Age ≥ 60 years
0.98 (0.74-1.29)
Age < 60 years
1.05 (0.48-2.27)
Male
0.99 (0.68-1.45)
Female
0.99 (0.68-1.45)
White
0.64 (0.26-1.53)
Black
1.38 (0.93-2.05)
Asian
1.25 (0.3-5.3)
Hispanic
1.34 (0.32-5.64)
Non-Hispanic
0.85 (0.58-1.23)
Income: 1st Quartile
1.08 (0.54-2.16)
Income: 2nd Quartile
0.8 (0.47-1.36)
Income: 3rd Quartile
1.15 (0.74-1.76)
Income: 4th Quartile
1.12 (0.64-1.97)
Medicare
0.61 (0.39-0.94)
Medicaid
1.27 (0.65-2.5)
Private
1.38 (0.94-2.04)
Self-pay
0.32 (0.04-2.34)
Hypertension
1.14 (0.88-1.47)
Type-2 Diabetes
1.14 (0.85-1.51)
COPD
0.53 (0.28-1)
Cardiovascular Diseases
0.85 (0.62-1.17)
CKD
0.94 (0.67-1.31)
Asthma
1.4 (0.84-2.33)
Vaccianted
1.06 (0.76-1.49)
Unvaccianted
1.06 (0.65-1.72)
0.40
1.0
2.0
3.00
5.00

B)

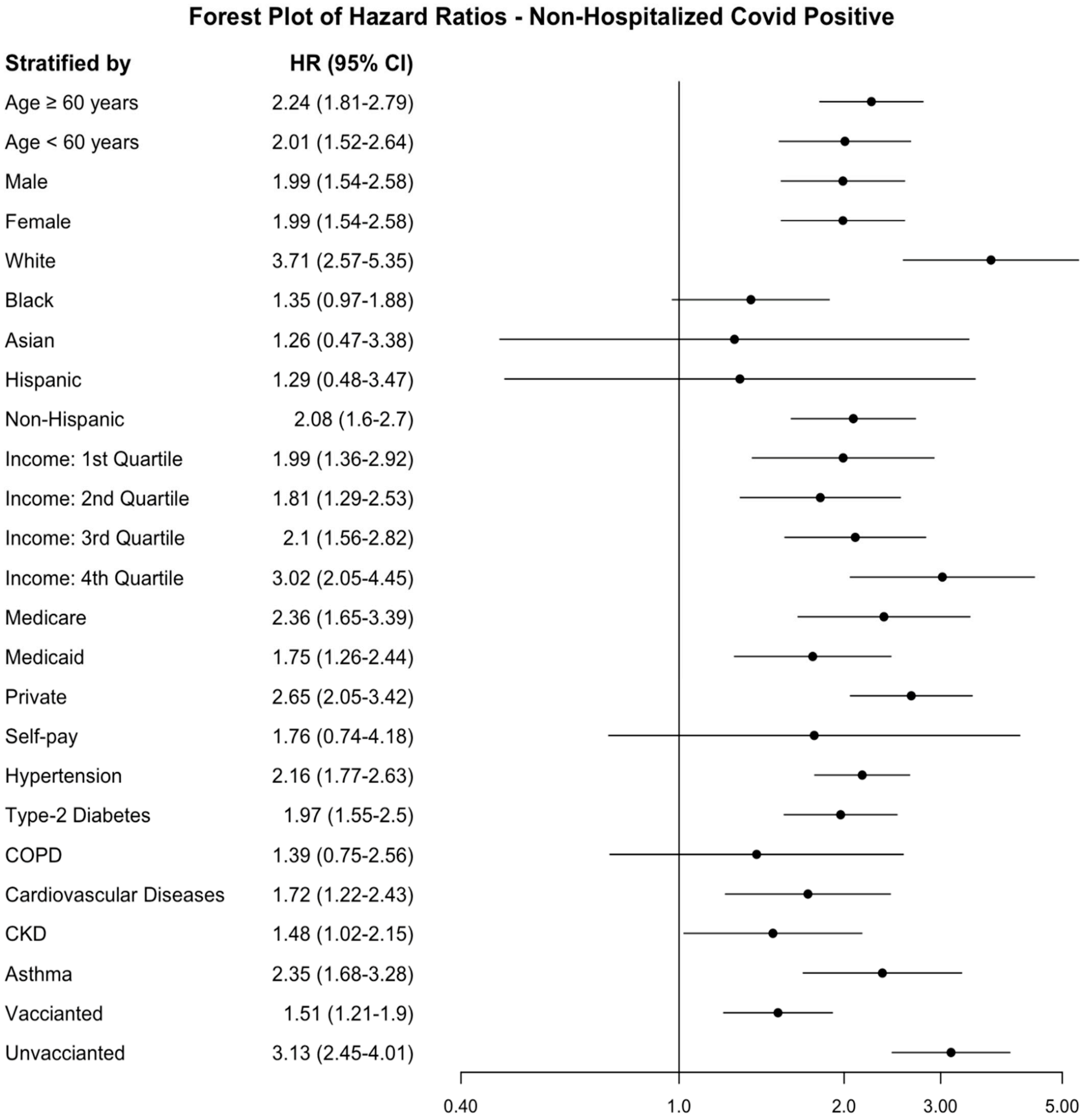
Forest Plot of Hazard Ratios - Non-Hospitalized Covid Positive
Stratified by
HR (95% CI)
Age ≥ 60 years
2.24 (1.81-2.79)
Age < 60 years
2.01 (1.52-2.64)
Male
1.99 (1.54-2.58)
Female
1.99 (1.54-2.58)
White
3.71 (2.57-5.35)
Black
1.35 (0.97-1.88)
Asian
1.26 (0.47-3.38)
Hispanic
1.29 (0.48-3.47)
Non-Hispanic
2.08 (1.6-2.7)
Income: 1st Quartile
1.99 (1.36-2.92)
Income: 2nd Quartile
1.81 (1.29-2.53)
Income: 3rd Quartile
2.1 (1.56-2.82)
Income: 4th Quartile
3.02 (2.05-4.45)
Medicare
2.36 (1.65-3.39)
Medicaid
1.75 (1.26-2.44)
Private
2.65 (2.05-3.42)
Self-pay
1.76 (0.74-4.18)
Hypertension
2.16 (1.77-2.63)
Type-2 Diabetes
1.97 (1.55-2.5)
COPD
1.39 (0.75-2.56)
Cardiovascular Diseases
1.72 (1.22-2.43)
CKD
1.48 (1.02-2.15)
Asthma
2.35 (1.68-3.28)
Vacciantedv
1.51 (1.21-1.9)
Unvacciantedv
3.13 (2.45-4.01)
0.40
1.0
2.0
3.00
5.00
